# Identification of Genetic Loci in *Lactobacillus plantarum* That Modulate the Immune Response of Dendritic Cells Using Comparative Genome Hybridization

**DOI:** 10.1371/journal.pone.0010632

**Published:** 2010-05-13

**Authors:** Marjolein Meijerink, Saskia van Hemert, Nico Taverne, Michiel Wels, Paul de Vos, Peter A. Bron, Huub F. Savelkoul, Jolanda van Bilsen, Michiel Kleerebezem, Jerry M. Wells

**Affiliations:** 1 Top Institute Food and Nutrition, Wageningen, The Netherlands; 2 Host-Microbe Interactomics, Wageningen University, Wageningen, The Netherlands; 3 NIZO Food Research, Ede, The Netherlands; 4 Pathology and Medical Biology, University Medical Centre Groningen, Groningen, The Netherlands; 5 Cell Biology and Immunology Group, Wageningen University, Wageningen, The Netherlands; 6 TNO Quality of Life, Zeist, The Netherlands; 7 Laboratory for Microbiology, Wageningen University, Wageningen, The Netherlands; National Institutes of Health, United States of America

## Abstract

**Background:**

Probiotics can be used to stimulate or regulate epithelial and immune cells of the intestinal mucosa and generate beneficial mucosal immunomodulatory effects. Beneficial effects of specific strains of probiotics have been established in the treatment and prevention of various intestinal disorders, including allergic diseases and diarrhea. However, the precise molecular mechanisms and the strain-dependent factors involved are poorly understood.

**Methodology/Principal Findings:**

In this study, we aimed to identify gene loci in the model probiotic organism *Lactobacillus plantarum* WCFS1 that modulate the immune response of host dendritic cells. The amounts of IL-10 and IL-12 secreted by dendritic cells (DCs) after stimulation with 42 individual *L. plantarum* strains were measured and correlated with the strain-specific genomic composition using comparative genome hybridisation and the Random Forest algorithm. This *in silico* “gene-trait matching” approach led to the identification of eight candidate genes in the *L. plantarum* genome that might modulate the DC cytokine response to *L. plantarum*. Six of these genes were involved in bacteriocin production or secretion, one encoded a bile salt hydrolase and one encoded a transcription regulator of which the exact function is unknown. Subsequently, gene deletions mutants were constructed in *L. plantarum* WCFS1 and compared to the wild-type strain in DC stimulation assays. All three bacteriocin mutants as well as the transcription regulator (lp_2991) had the predicted effect on cytokine production confirming their immunomodulatory effect on the DC response to *L. plantarum*. Transcriptome analysis and qPCR data showed that transcript level of *gtcA3*, which is predicted to be involved in glycosylation of cell wall teichoic acids, was substantially increased in the *lp_2991* deletion mutant (44 and 29 fold respectively).

**Conclusion:**

Comparative genome hybridization led to the identification of gene loci in *L. plantarum* WCFS1 that modulate the immune response of DCs.

## Introduction

Several species of *Lactobacillus* are naturally present in the human intestinal tract and several species and strains have been evaluated for their probiotic activity. Oral administration of certain probiotic strains has shown significant and promising results in human clinical trials and experimental animal models of inflammatory bowel disease, irritable bowel disease and allergy [1], [2], [3], [4], [5], [6], [7], [8]. Conclusive evidence for the mechanisms underlying the beneficial properties of probiotics is lacking but results obtained from *in vitro* studies and animal intervention models indicate a strong role for immunomodulation and enhancement of the epithelial barrier functions [9], [10]. Proposed immunomodulatory mechanisms include down-regulation of inflammatory responses through the modulation of dendritic cell (DC) function and subsequent expansion or induction of regulatory T cells producing anti-inflammatory cytokines TGF-beta and IL-10 [11], [12]. The capacity of lactobacilli to stimulate pro-inflammatory or anti-inflammatory cytokines in co-culture with DCs or PBMCs indicates that different strains of *Lactobacillus plantarum* may differentially stimulate immune cells and could therefore exert opposite immunomodulatory effects.

Dendritic cells play a key role in mucosal immunity and tolerance and not surprisingly have been implicated as key players in the interaction between probiotics and the immune system. In the small intestine DCs are known to sample microbes that enter the Peyer's Patches via M-cells [13] but also directly across the epithelium by opening tight junctions and sending dendrites to the luminal side [14]. In the mucosa DCs are the main activators of naive T cells and their T cell polarising properties are largely governed by the nature of the microbial products encountered at mucosal sites. Additionally cellular cytokines such as TSLP and TGF-beta which are produced by epithelial cells can also modulate the function of resident DCs [15], [16]. Previous studies have shown that the DC responses to different probiotic bacteria are strain-specific and that this can have different outcomes on T cell polarisation [17], [18], [19], [20], [21]. Immature DCs are characterized by a high capacity for antigen uptake and following antigen capture in the presence of appropriate stimuli, they migrate to secondary lymphoid organs and mature. During maturation the major histocompatibility complex (MHC) molecules for antigen presentation and of co-stimulatory molecules, such as CD40, CD54, CD83 and B7.1 and B7.2 (CD80 and CD86) are up regulated for an effective T-cell stimulation [22]. T helper(h) 1 immune responses critically depend on the ability of DCs to produce interleukin (IL)-12 and are characterized by the production of interferon (IFN)-gamma and IL-2, which induce cell-mediated immunity. Th2 immune responses involve IL-4, IL-5, IL-6, and IL-13 and induce humoral immunity. IL-10 is a critical regulatory T cell cytokine which suppresses IL-12 production and consequently other Th1 cytokines such as IFN-gamma and tumor necrosis factor alpha (TNF-alpha). It also prevents activation of antigen-presenting cells, inhibits the maturity of DC, limits T-cell proliferation, and can induce a state of antigen-specific tolerance. In the gut, IL-10 is a key molecule for the induction of T regulatory cells and prevention of mouse colitis [23], [24], [25]. The suppression of IL-12 in the gut by IL-10 prevents an inflammatory cascade of Th1 cytokines and cellular migration. The ability of different lactobacilli to induce a high ratio of IL-10/IL-12 production in human peripheral blood mononuclear cells (PBMCs) has been shown to correlate with their capacity to provide significant protection from TNBS induced colitis in mice [26].

Lactobacilli have been shown to differ substantially in their ability to modulate DC responses and subsequent T cell induction but the factors responsible for these differences remain largely unknown. Comparative genomic analysis is one approach to identify genetic loci linked to certain phenotypes and has been applied to *Lactobacillus spp.* to identify a genes involved persistence in the intestine [27], mannose binding [28], and pilli formation [29]. Several innate pattern recognition receptors (PRR) recognizing evolutionary conserved microbe-associated molecular patterns (MAMPS) are potentially involved in immunomodulation by lactobacilli. The most well characterised family of innate receptors are the Toll-like receptors (TLR) of which TLR2 is known to recognise bacterial cell wall components such as peptidoglycan (PG), and when present as a heterodimer with TLR6 can also recognize the fatty acid chains present on liopteichoic acid (LTA) [30], [31]. There are considerable differences between the LTA molecules of different *Lactobacillus* species and strains which may influence their immune stimulating activities, including variation of the quantity of LTA produced per cell, its chain length, and its degree of substitution with D-alanine and/or glucose [32], [33], [34]. For example, a D-alanyl transfer protein B (*dltB*) deletion mutant of *L. plantarum* induced higher levels of the anti-inflammatory cytokine IL-10 in PBMC co-culture experiments than the wild type strain thereby increasing its protective effect in a TNBS mouse model of intestinal colitis [30]. *DltB* is a putative transmembrane protein predicted to be involved in the passage of the activated d-alanyl-Dcp complex across the glycerol phosphate backbone of LTA. Thus, the altered phenotype of the *dltB* mutant was attributed to the loss of D-alanyl substitution in LTA although an increase in glucose substitution on LTA was also observed. In *L. rhamnosus* the DltD membrane protein facilitates the binding of Dcp for ligation with d-Ala and additionally has thioesterase activity for removing mischarged d-alanyl carrier proteins. In contrast to *L. plantarum*, deletion of the D-alanyl transfer protein in *L. rhamnosus* had no effect on cytokine production in co-culture with PBMCs [35]. These findings suggest the involvement of multiple interacting bacterial molecules in the observed phenomena. TLR2 in combination with TLR1 or TLR6 can also recognise the fatty acid chains present on lipidated lipoproteins of bacteria although for Lactobacillus this topic remains relatively unexplored.

Extracellular polysaccharides (EPS) are potentially important probiotic effector molecules and they show a high level of diversity and complexity among different strains and species. EPS could have a shielding effect on the recognition of LTA by host receptors or a direct effect on immunomodulation by binding to specific lectin receptors such as the dendritic-specific intercellular adhesion molecule 3-grabbing non intergrin DC-SIGN [36]. Yasuda and colleagues have shown that in *Lactobacillus casei* the cell wall associated polysaccharide PS-1 functions as a modulator that suppresses the immune response of macrophages and monocytes [37].

The NOD-like receptors NOD1 and NOD2 are intracellular receptors for muropeptides of the PG in the bacterial cell wall. NOD2 recognises muramyl dipeptide which is present in lactobacilli but for these peptides to be released PG would have to be hydrolysed by PG-hydrolases of either bacterial or host origin (lysozyme). An immune evasion strategy established for some pathogens involves the action of cell wall modifying enzymes to prevent cell wall degradation by lysozyme and thus the release of immunostimulatory muropeptides [38], [39].

The aim of this study was to identify genetic loci that encode, modify or regulate immunomodulating factors in *L. plantarum* by measurement of cytokine responses to 42 different *L. plantarum* strains and a comparative genome hybridization approach based on the genome sequence for *L. plantarum* WCFS1 and comparative genome hybridization datasets of different *L. plantarum strains* [40]. WCFS1 is a single colony isolate of strain NCIMB8826, which was originally isolated from human saliva and known to survive gastrointestinal passage after oral administration in healthy volunteers [41]. Host responses to *L. plantarum* WCFS1 were recently demonstrated in health human volunteers by transcriptome analyses of duodenal tissue samples [42], [43]. Mutation analysis of several of these candidate genes in *L. plantarum* WCFS1 allowed us to evaluate their immunomodulatory properties and had the predicted effect on cytokine production confirming their immunomodulatory effect on the DC response to *L. plantarum*.

## Materials and Methods

### Bacterial strains

42 different *L. plantarum* strains isolated from humans and different food resources were used for immunoassays and comparative genome hybridization studies (CGH) (Table S1). Genomic composition of the strains has been previously described [44], [45]. *L. plantarum* strains were grown overnight to stationary phase at 37°C in Mann-Rogosa Sharpe (MRS) broth (Merck, Darmstadt, Germany). The bacteria were recovered by centrifugation and washed twice in phosphate buffered saline (PBS, pH = 7.4) and resuspended at 2×10^8^ CFU/ml in PBS containing 20% glycerol and stored at −80°C prior to use in the immunoassays. Colony forming units (CFU) were determined by plating serial dilutions of the cultures on MRS agar.

### Blood donors

The study was approved by Wageningen University Ethical Committee and was performed according to the principles of the Declaration of Helsinki. Buffy coats from blood donors were obtained from the Sanquin Blood bank in Nijmegen (The Netherlands). A written informed consent was obtained before the sample collection.

### Differentiation and maturation of dendritic cells

Human monocytes were isolated from blood using a combination of Ficoll density centrifugation and cell separation using CD14-specific antibody coated magnetic microbeads (Miltentyi Biotec). The purity of isolated CD14+ cell fraction was greater than 90% in all experiments. To generate immature DC (iDCs), the purified CD14+ cells were cultured for 6 days in the presence of IL-4 (R&D systems) and GM-CFS (R&D systems) to differentiate into myeloid dendritic cells. At day 6 the cells were left unstimulated (iDCs) or were stimulated with LPS (1 µg/mL) or with different *L. plantarum* strains or WCFS1 deletion mutants (1∶1 bacteria to DC ratio) for 48 hours. As anticipated, as a consequence of the application of antibiotics, no bacterial growth was observed during this period.

### Analysis of cell surface markers and measurement of cell death by flow cytometry

During the 8 day culture period of the CD14+ cells, cells were stained on days 3, 6 and 8 with fluorescence-conjugated monoclonal antibodies specific for CD83, CD86 or their isotype-matched controls (BD biosciences, San Diego, USA) and analyzed by flow cytometry (FACSCanto II, BD, San Diego, USA). To check the activation status of the cells (data from day 3 and 6 not shown), the CD86 expression on the cells were measured. CD83 was not expressed on immature dendritic cells (d3 and 6) but was highly expressed on DC after activation with known maturation factors (e.g. LPS)[46]. Live, apoptotic and necrotic cells were discriminated by staining with Annexin V and propidium iodide on days 3, 6 and 8 according to the manufacturer's protocol. The cells were analyzed on a flow cytometer (FACSCanto II, BD, San Diego, USA). Cells that are negative for both Annexin V and PI are not apoptotic or necrotic as translocation of the membrane phospholipid phosphatidylserine has not occurred and the plasma membrane is still intact. Therefore, Annexin V and PI double negative cells were considered as viable cells, whereas both single and double positive cells were regarded as non-viable [47]. The flow cytometry data was analyzed using the BD FACSDiva software. On days 3 to 8 the viability of the cells was between 50–80%.

### Cytokine analysis

Supernatants from the DC stimulation assays were collected after stimulation for 48 hours and analyzed for the presence of cytokines (TNF-alpha, IL-12p70 and IL-10) using a cytometric bead-based immunoassay that enables multiplex measurements of soluble cytokines in the same sample [48], according to the manufacturer's protocol (BD biosciences). The limits of sensitivity for detection were as follows: TNF-alpha 0.7 pg/mL; IL-12p70 0.6 pg/mL and IL-10 0.13 pg/mL. The antibody used to measure IL-12 recognizes the dimeric cytokine designated IL-12p70 whereas some antibodies recognize the p40 subunit which is also present in IL-23. The flow cytometry data were analysed using the BD FCAP software.

### Identification of candidate genes involved in cytokine secretion by gene-trait matching

Candidate *L. plantarum* genes, that were potentially involved in modulation of the DC responses were identified by *in silico* gene-trait matching [28] using genotype information referenced from the *L. plantarum* WCFS1 genome [40], [44]. The significance of the gene-trait co-occurrence was assessed by assuming a discrete probability distribution of genes and traits in the context of a null hypothesis that co-occurrence is caused by a random process [49]. All *L. plantarum* genes were tested for their significant co-occurrence with each cytokine concentration or cytokine concentration ratio (i.e. IL-10/IL-12). *L. plantarum* WCFS1 genes with the highest variable importance measures as returned by the Random Forest method were selected for further characterization using a gene deletion approach in combination with immunoassays.

### Construction of knock-out mutants

**Figure 1:**
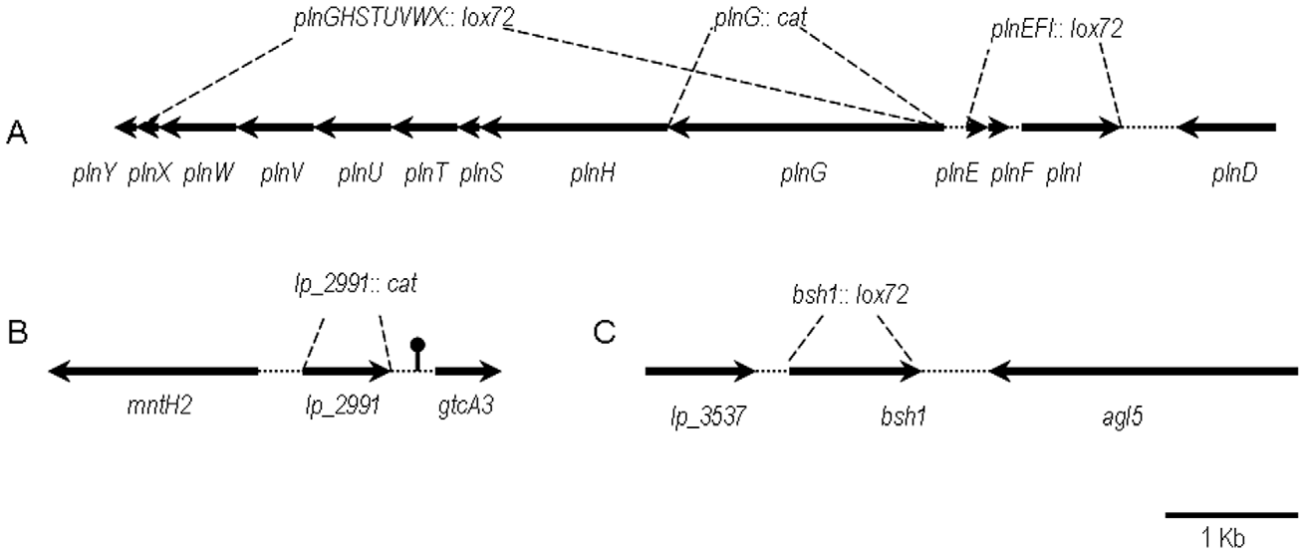
Schematic representation of the genetic loci targeted by mutagenesis in *L. plantarum* WCFS1. Each gene is indicated by an arrowed line the length of which is indicated by the scale bar. Dotted lines show the chromosomal genes replaced by the choramphenicol acetyl transferase gene cassette (*cat*) or deleted using the cre-lox system leaving an insertion of the lox72 sequence (*lox72*). The transcription terminator identified downstream of *lp-299*1 is indicated by a ball and stick. A. The bacteriocin locus. B. Genes adjacent to the putative transcription regulator *lp-2991*. C. Genes adjacent to the bile salt hydrolase gene *bsh1*.

A previously described *L. plantarum lp_3536* deletion mutant was used in this study [50]. Gene deletion mutants of *lp_0419-0422* and *lp_0423-30* were constructed for a different study involving immune cytokine profiling of lactobacilli with peripheral blood mononuclear cells (van Hemert et al., personal communication) (Fig. 1). Construction of the *L. plantarum* gene deletion mutants for *lp_0423* and *lp_2991* was performed as previously described with several modifications [51]. The upstream and downstream flanking regions of the target genes, as well as the *lox-cat-lox* region of pNZ5319 were amplified by PCR. The resulting amplicons were used as templates in a SOEing PCR reaction [52] linking the flanking regions and lox-cat-lox together by means of complementary 5′ regions in the primers (Table 1). Subsequently, PCR products were cloned into the non-replicating integration vector pNZ5319 [51] digested with SwaI and Ecl136II. Plasmids were transformed into competent cells of *E. coli* JM109 by electroporation as described by the manufacturer (Invitrogen). This resulted in a plasmid containing the complete gene replacement cassette. Plasmid DNA was isolated from *E. coli* by using Jetstar columns, following the manufacturer's instructions (Genomed GmbH, Bad Oeynhausen, Germany). The sequence of the cloned DNA was confirmed by sequence analysis (BaseClear, Leiden, The Netherlands).

**Table 1:** Primers used for deletion mutants in *L. plantarum* WCFS1 and RT-PCR.

Primer	Sequence^a^
LF2991F	5′- CCGTTTACTGAACGACTTGTCG-3′
LF2991R	5′- **CGAACGGTAGATTTAAATTGTTT** TGAAAAATTCATTTTCACACCTCC -3′
RF2991F	5′- **GTACAGCCCGGGCATGAG**AAGACTTCAGATTAGGTGTTCAG -3′
RF2991R	5′- TACTCGTCATTCTAACTACCGC-3′
Ecl-loxR	5′- AAACAATTTAAATCTACCGTTCG -3′
Pml-loxF	5′- CTCATGCCCGGGCTGTAC -3′
LF2991F2	5′-TGGCACCGATAATCCCTAAAGC-3′
RF2991R2	5′-TGTAATCTTAATCCGCTTTCACAC-3′
LF423F	5′-AATTGATACATGTGGTTTCGAAAG-3′
LF423R	5′- **CGAACGGTAGATTTAAATTGTTT**CCAATGCATACTTGTACTCCC -3′
RF423F	5′- **GTACAGCCCGGGCATGAG**CGACTTGATCAATAGCTGAGGG-3′
RF423R	5′-TTGGTTGCCTTGATCGTGTAAG-3′
Tr-F (transcription regulator lp_2991)	5′-CAAACTGACATCGCGACAC-3′
Tr-R	5′-CGTAATAACCCCCACTAATTG-3′
mntH2-F (manganese transport protein)	5′-TTGTGGATTTTAACCGAATTAG-3′
mntH2-R	5′-TCTTCCGCGTTTGTGAGA-3′
gtcA3-F (teichoic acid glycosylation protein)	5′-ATGAGTAAGCCAGATTCGATTA-3′
gtcA3-R	5′-GCCGAGCTCCCCAATA-3′
recA-F (recombination protein RecA)	5′-TGGATCGTTGGCCTTAGA-3′
recA-R	5′-CCGGAGCGCTTGTGA-3′

a Bold and underlined nucleotides indicate overlapping ends with the Ecl-loxR and Pml-loxF primers.

A double cross-over gene replacement strategy was used to substitute the target gene(s) with a chloramphenicol gene (*cat*). The mutagenesis plasmids were transformed to *L. plantarum* WCFS1 and integrants were selected on MRS plates containing 10 µg/mL chloramphenicol. After growth at 37°C for 2 to 4 days, full-grown colonies were replica-plated to MRS with or without 30 µg/mL erythromycin. Colonies displaying an erythromycin sensitive phenotype represent candidate *cat*-gene replacements resulting from a simultaneous double cross-over event in both the 5′- and 3′-flanking regions of the target protein. The anticipated *cat*-replacement genotype was confirmed by PCR using primers flanking the sites of recombination (Table 1). One integrant displaying the anticipated phenotype and genotype was selected for all mutants and was utilized in the remainder of the study.

### RNA isolation

Bacteria (WCFS1 and delta-lp_2991) were grown in MRS to OD_600 nm_ of 1 and then 40 mL of quenching buffer (66.7 mM HEPES (pH 6.5) 60% methanol, −40°C) was added to 10 mL of bacterial suspension to quench cellular processes. Cells were harvested by centrifugation at 15500 g for 10 min at −20°C and then resuspended in 400 µL of ice-cold MRS. The cell suspensions were transferred to ice-cold tubes containing 0.5 g of glass beads, 400 µL of phenol, 100 µL chloroform, 30 µL 10% sodium dodecyl sulfate and 30 µL 3 M sodium acetate (pH 5.2). Cells were disrupted with three 40-s treatments in a Fastprep (Obiogene Inc. Illkirch, France) separated by 1 min intervals on ice. After centrifugation the aqueous phase was used for RNA isolation with a High Pure kit (Roche diagnostics, Mannheim, Germany). The RNA concentration was measured at an OD of 260 nm with the ND-1000 spectrophotometer (NanoDrop Technologies Inc.). The *A*_260_/*A*_280_ ratio was measured to check purity of the RNA. Quality of the RNA obtained was analyzed with the 2100 Bioanalyser (Agilent Technologies). Samples whereby the 23S/16S RNA ratio was superior or equal to 1.6 were taken for further labeling.

### cDNA synthesis and quantitative reverse transcription-PCR

cDNA was synthesized from 1 µg of total purified bacterial RNA and random oligonucleotide hexamers using Superscript III reverse transcriptase (Invitrogen, Breda, The Netherlands). Reverse transcription was performed at 55°C for 60 min, after which the reverse transcriptase was inactivated at 85°C for 5 min. Primers were designed using Oligo Program version 6 (MedProbe, Oslo, Norway) to have melting temperatures between 60 and 62°C and an amplicon size of approximately 400 bp (Table 1). Quantitative PCR was performed with the synthesized cDNAs by using Absolute SYBR Green QPCR (Westburg). Each reaction mixture contained 1x SYBR Green master mix, 70 nM of each primer and 5 ng of reverse-transcribed RNA. After activation of Thermoprime Plus DNA polymerase at 95°C for 15 minutes, PCR was carried out for 40 cycles of 95°C for 15 s, 55°C for 30 s and 72°C for 30 s. The identities of the amplicons resulting from the reactions were checked after amplification by melting curve analysis and 1.5% agarose gel electrophoresis. Reaction mixtures containing no template were included in each real-time PCR experiment to control for contamination. Relative transcript levels were calculated by using the comparative ΔΔ*Ct* method described by Pfaffl et al. [53]. The average *Ct* values observed for the target gene transcripts (lp_2991, mntH2 and gtcA3) were normalized to the average *Ct* values obtained for the reference gene recA from the same RNA sample. Two replicates of all samples and primer pairs were included and the experiment was performed in duplicate.

### Transcriptome analysis

A two-color microarray-based gene expression analysis was performed on a custom-made 60-mer oligonucleotide array (Agilent Biotechnologies, submitted in GEO under platform GPL9359) to determine the global gene transcription levels of WCFS1 and the lp_2991 deletion mutant. Cy3- and Cy5-labeled cDNAs were prepared using a Cyscribe post labeling kit (GE Healthcare, United Kingdom). Slides were prehybridized for 45 min at 42°C in 20 ml prehybridization solution (1% bovine serum albumin, 5× SSC, 0.1% sodium dodecyl sulfate; filtered) (1× SSC is 0.15 NaCl plus 0.015 M sodium citrate), washed in filtered deionized water, and dried. Co-hybridization with Cy5- and Cy3-labeled cDNA probes was performed overnight at 42°C for 16 h in Slidehyb#1 (Ambion, Austin, TX). The slides were then washed twice in 1× SSC-0.1% sodium dodecyl sulfate and twice in 1× SSC before they were scanned. The experiment was performed with Cy5/Cy3 dye swaps.

Slides were scanned with a ScanArray Express 4000 scanner (Perkin Elmer, Wellesley, MA), and image analysis and processing were performed using the ImaGene Version 7.5 software (BioDiscovery Inc., Marina Del Rey, CA, USA). The microarrays were scanned at different intensities. For each of the individual microarrays the best scan was selected on the basis of signal distribution (combination of a low number of saturated spots and a low number of low signal spots). The data were normalized using Lowess normalization as available in MicroPrep [54]. The data were corrected for inter-slide differences on the basis of total signal intensity per slide using Postprep[54]. The median intensity of the different probes per gene was selected as the gene expression intensity. CyberT was used to compare the different transcriptomes, taking into account the duplicates (dye swaps) of each of the conditions [55]. This analysis resulted in a gene expression ratio and false discovery rate (FDR) for each gene. Genes with FDR values <0.05 were considered to be statistically significant. All microarray data is MIAME compliant and are available in GEO.

### Statistical analysis

Mixed general linear model using restricted maximum likelihood (REML) was used to determine the statistical differences within donors between cytokine produced by DCs stimulated with the constructed deletion mutants compared to the wild type *L. plantarum* WCFS1. A two-sided *p*-value of 0.05 or lower was considered to be significant. The statistical analysis was performed by using SAS software (version 9.1, SAS Institute Inc., Cary, NC, USA).

## Results

### DC cytokine responses to different *L. plantarum* strains

**Figure 2:**
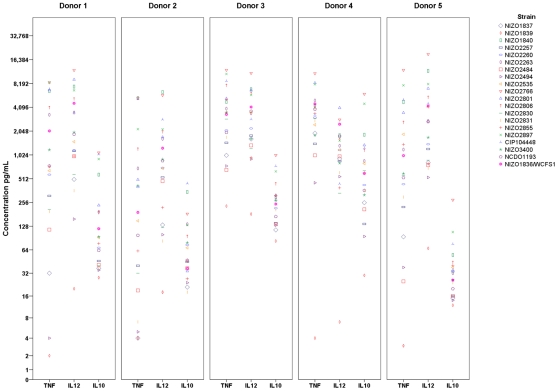
Cytokine secretion by dendritic cells. IL-10, TNF-alpha and IL-12p70 production by monocyte-derived dendritic cells derived from blood of five different donors after stimulation with 20 different *L. plantarum* strains. Each symbol represents a different *L. plantarum* strain.

Monocyte derived immature dendritic cells were cultured in the presence of 42 different *L. plantarum* strains. Based on the variation in CD83 and CD86 expression and cytokine secretion, 20 strains were selected to repeat the culturing with monocyte derived DCs from 5 different donors. The strains differed considerably in their ability to modulate DC responses (Fig. S1 and Fig. 2). For example, the amounts of IL-10 induced by the strains ranged from 28 pg/mL to 1095 pg/mL (39 fold) and for IL-12 the values ranged from 20 to 11996 pg/mL (600 fold). For TNF-alpha some strains induced very low amounts (close to the detection limit of 0.7 pg/mL) whereas others induced 8.4 to 12 ng/mL (Fig. 2 and Table 2). The large variation in strain immune profiles suggested that there could be some underlying strain-dependent genetic differences influencing the innate response to *L. plantarum*.

**Table 2:** Ranges of DC cytokine induction by *L. plantarum* strains.

		Range*	Fold difference*	Highest strain	Lowest strain
**Donor 1**	**IL-10**	(35–1095)	31	NIZO2766	NIZO2494
	**IL-12**	(158–11996)	76	NIZO2766	NIZO2494
	**TNF-alpha**	(4–8412)	2103	NIZO2897	NIZO2494
**Donor 2**	**IL-10**	(18–447)	25	CIP104448	NIZO2831
	**IL-12**	(83–6358)	77	LMG18021	NIZO2831
	**TNF-alpha**	(24–5352)	223	NIZO1840	NIZO1837
**Donor 3**	**IL-10**	(115–1017)	9	NIZO2766	NIZO1837
	**IL-12**	(909–11026)	12	NIZO2766	NIZO2494
	**TNF-alpha**	(663–12052)	18	NIZO2766	NIZO2484
**Donor 4**	**IL-10**	(95–6011)	63	NIZO2766	NIZO2494
	**IL-12**	(336–4026)	12	NIZO2801	NIZO2830
	**TNF-alpha**	(455–10945)	24	NIZO2766	NIZO2494
**Donor 5**	**IL-10**	(14–276)	20	NIZO2766	NIZO2494
	**IL-12**	(532–19114)	36	NIZO2766	NIZO2494
	**TNF-alpha**	(25–12052)	482	NIZO2766	NIZO2484

*NIZO1839 was excluded, since DC cytokine induction by strain NIZO1839 were strikingly lower than for all other strains.

Some strains such as NIZO2766, NIZO2801 and NIZO2897 were clearly strong inducers of pro-inflammatory cytokines IL-12 and TNF-alpha while others were considerably less potent (e.g. strains NIZO1839, NIZO2494 and NIZO2831; Fig. 2 and Table 2). Similarly, the strains showed strikingly different capacities to induce the anti-inflammatory cytokine IL-10. From a comparison of IL-12 to IL-10 ratios it is clear that these cytokines can vary independently of each other allowing the possibility for strains with distinct pro-inflammatory (e.g. strain NIZO1840 and NIZO2257) and anti-inflammatory profiles (e.g. strain CIP104448). As expected levels of cytokines induced by *L. plantarum* strains differed between donors but the ranking of the strains was highly consistent for each cytokine showing that the strain immunoprofiles were reproducible (Fig. 2).

### Identification of candidate gene loci by *in silico* gene trait matching

*L. plantarum* genes potentially involved in the production of pro and anti-inflammatory cytokines were identified by *in silico* gene-trait matching by correlating measurements of cytokines induced by the different strains with their genotypic information based on comparative hybridisation to a whole genome microarray [44], [45]. Seven genes displayed a match with lower levels of IL-10 concentration in the co-culture system. One of these genes, *lp_2991* is annotated as a transcription regulator which is present in 90% of the strains tested (Table 3). The other six genes (*lp_0422*, *lp_0423*, *lp_0424a*, *lp_0424*, *lp_0425* and *lp_0429*) lie within the plantaricin locus (*lp_0403* to *lp_0431*) involved in plantaricin biosynthesis and secretion. The *plnEFI* operon (l*p_0419* to *lp_0422*) is encoding two bacteriocin-like peptides and a bacteriocin immunity protein. Homologues of the gene loci in this operon are present in 81-85% of the tested strains. *Lp_0423* is distal to *lp_0422* and located in another operon and encodes an ABC transporter involved in the transport of bacteriocins [56], [57]. *Lp_0423* (*plnG*) is present in 88% of the tested strains [58].

**Table 3:** Candidate genes identified by gene trait matching.

Gene name	Gene nr	Description	Importance^a^	Predicted k.o. phenotype^b^
*Lp_2991*	lp_2991	Transcription regulator	6.4E06 (IL-10)1.5E08 (TNF-alpha)	IL-10 and TNF-alpha ↑
*plnEFI*	lp_0419-lp_0422	Bacteriocin like peptide EBacteriocin like peptide FImmunity protein plnI	1.1E07	IL-10 ↑
*plnG*	lp_0423	ABC transporter	2.4E06	IL-10 ↑
*plnGHSTUVWX*	lp_0423-30	Bacteriocin ABC-transporter, ATP-binding and permease protein plnG	3.6E06	IL-10 ↑
*bsh1*	lp_3536	choloylglycine hydrolase	3.4E07	TNF-alpha **↑**

a Estimated importance values as given by Random Forest.

b Phenotype in DC assay affected by the presence of the gene.

↑ indicated a higher effect when the gene is absent.

Three genes (*lp_2991*, *lp_0422* and *lp_3536*) had a gene-trait match with a lower concentration of TNF-alpha produced in the supernatant of *L. plantarum* DC co-culture (Table 3). There was a co-occurrence of low TNF-alpha secretion and the presence of *lp_2991* and *lp_3536*. *Lp_3536* is predicted to encode a bile salt hydrolase capable of removing the amino acid moiety from the steroid nucleus of conjugate bile salts by hydrolysis and is present in 81% of the tested strains.

### Validation of the role of the candidate genes in cytokine secretion

**Figure 3:**
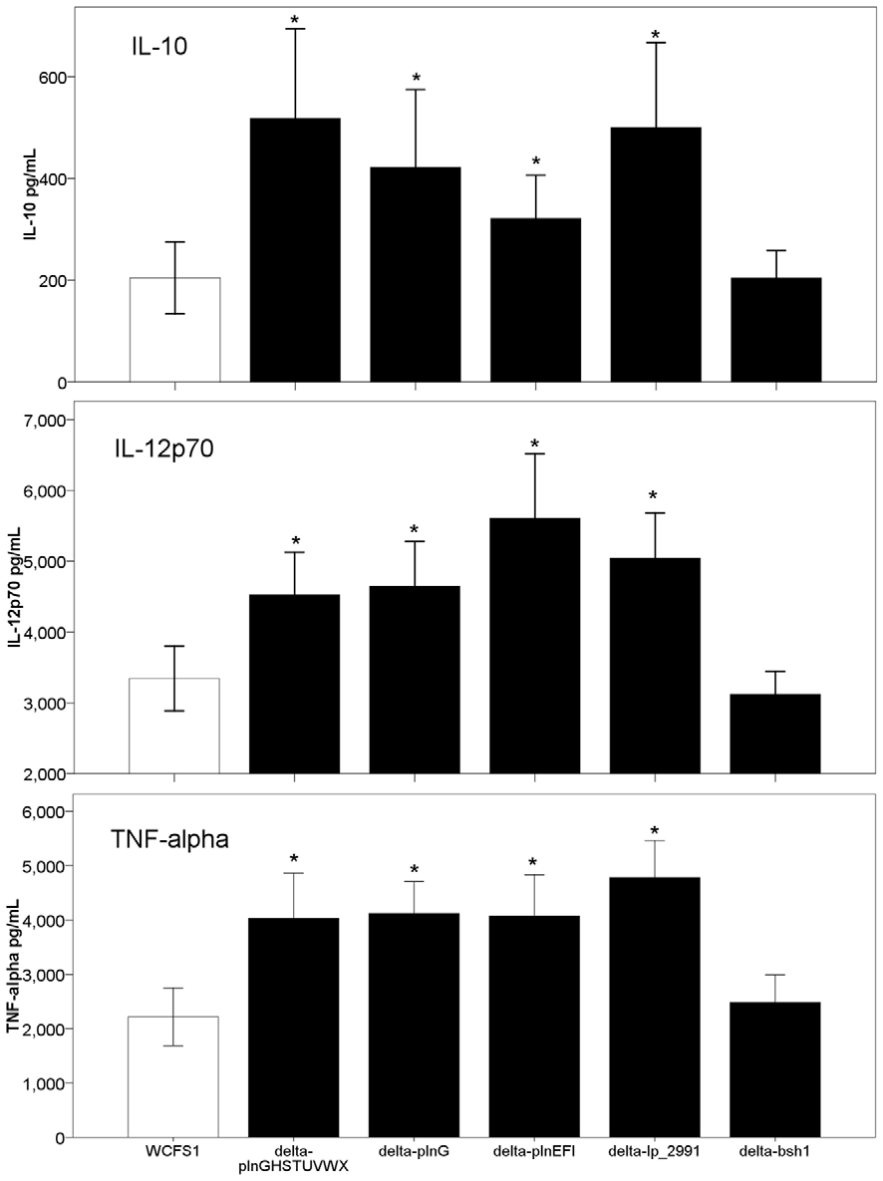
Validation of the candidate genes in different deletion mutants of *L. plantarum* WCFS1. IL-10, IL12p70, and TNF-alpha secretion by monocyte derived dendritic cells after stimulation with different *L. plantarum* deletion mutants and the wild type strain WCFS1. Data are presented as the mean +/− standard error of the mean from five donors of duplicate wells. Differences between wild-type and mutant were calculated according mixed general linear model using REML. A two-sided *p*-value of 0.05 or lower was considered to be significant indicated *.

To further investigate the role of the genes identified by gene-trait matching in modulating DC cytokine secretion, specific gene-replacement mutants of genes *lp_2991* and *lp_0423* were constructed in *L. plantarum* strain WCFS1, to yield strains *lp_2991*::*cat* and *plnG::cat*. Construction of the *bsh1::lox72*, *plnGHSTUVWX::lox72* and *plnEFI:lox72* mutant is described in the Material and Methods section above. The capacity of the deletion mutants to induce IL-10, IL-12p70 and TNF-alpha production by DCs was higher than compared to the wild-type strain *L. plantarum* WCFS1. As predicted on basis of the gene-trait correlation, deletion mutants lacking genes involved in plantaricin secretion and immunity induced significantly higher amounts of IL-10 in DC co-culture compared to the wild type strain WCFS1 (Fig. 3 and Table 4). For mutant *plnEFI::lox72* in which the two bacteriocin-like peptides and a bacteriocin immunity protein were deleted, IL-10 was significantly increased 3.3 fold (p<0.05). Deletion of the pheromone and bacteriocin transport operon (*plnGHSTUVWX*), in strain *plnGHSTUVWX::lox72* also significantly increased IL-10 3.1-fold (p<0.05) compared to the wild type strain WCFS1. Similar increases (3.2 –fold; p<0.05) were also observed for *plnG::cat* (Fig. 3 and Table 4). In the *plnEFI*, *plnGHSTUVWX* and *plnG* mutants TNF-alpha secretion was significantly increased by 4.2-fold (p<0.05), 4.1-fold (p<0.05) and 7.4 fold (p<0.05) respectively. In all plantaricin associated mutants IL-12p70 secretion was also significantly (p<0.05) increased between 1.9 and 2.4 fold.

**Table 4:** Validation of the candidate genes knock out mutants.

Gene name	Gene nr	Predicted k.o. phenotype^a^	p-value IL-10^b^	p-value TNF-alpha^b^	p-value IL-12p70^b^
*lp_2991*	lp_2991	IL-10 and TNF-alpha ↑	0.0016	0.0058	0.0003
*plnEFI*	lp_0419-lp_0422	IL-10 ↑	0.0064	0.0095	0.0001
*plnG*	lp_0423	IL-10 ↑	0.0024	0.0144	0.0117
*plnGHSTUVWX*	lp_0423-30	IL-10 ↑	0.0003	0.1041	0.0016
*bsh1*	lp_3536	TNF-alpha **↑**	0.2449	0.6648	0.6431

a Phenotype in DC assay affected by the presence of the gene.

↑ indicated a higher effect when the gene is absent.

b Difference between wild-type and mutant according mixed general linear model using REML. A two-sided *p*-value of 0.05 or lower was considered to be significant.

The presence of the *lp_2991* gene in strains was associated with induction of lower amounts of IL-10 and TNF-alpha secretion compared to strains lacking this gene. As predicted by gene-trait correlation analysis, deletion of this gene in wild type strain WCFS1 significantly increased IL-10 and TNF-alpha secretion compared to the wild type strain. IL-10 secretion was increased 6.3-fold (p<0.05) and TNF-alpha secretion was increased 17.2-fold (p<0.05). Additionally, IL-12p70 secretion was induced 3.2-fold (p<0.05) (Fig. 3 and Table 4). Deletion of *lp_3536* (strain *lp_3536::loxp72*) had no significant effect on cytokine production compared to the wt strain (Fig. 3 and Table 3). In conclusion four of the five deletion mutants showed the predicted effect on DC immune responses.

### Transcriptome analysis

Transcriptomes of the WCFS1 and lp_2991 deletion mutant were compared to obtain an expression ratio for each gene. Genes with FDR values <0.05 were considered significant. Of the 3100 genes present on the microarray 25 genes were significantly upregulated in the lp_2991 deletion mutant and 33 genes were significantly downregulated (Table S2).

In the lp_2991 deletion mutant, the gene lp_2991 signal was not significantly different to the wild type due to the very low expression (signal) level. In the mutant the most highly increased transcript (44.7-fold) was the *gtcA3* gene which is predicted to be involved in glycosylation of cell wall teichoic acids including LTA which is an agonist of TLR2. Another gene associated with cell envelope structures that showed increased expression in the mutant was tagO (4.4-fold), encoding an enzyme involved teichoic acid synthesis. A group of 6 genes involved in nucleoside and nucleotide synthesis were 4.1 to 17 fold higher in the mutant as was the *purR1* pyrimidine operon regulator (2.6 fold). The reasons for this are not clear but these genes are regulated by several factors and are often differentially expressed in microarray experiments (unpublished results Michiel Kleerebezem). Transcript levels of several genes encoding hypothetical proteins and transporters were either increased or decreased in the mutant compared to the wild type. In the mutant, the largest decrease in expression was for *lp_2531* which is predicted to be a PTS transporter for N-acetylglucosamine and glucose.

### RT-PCR results

**Figure 4:**
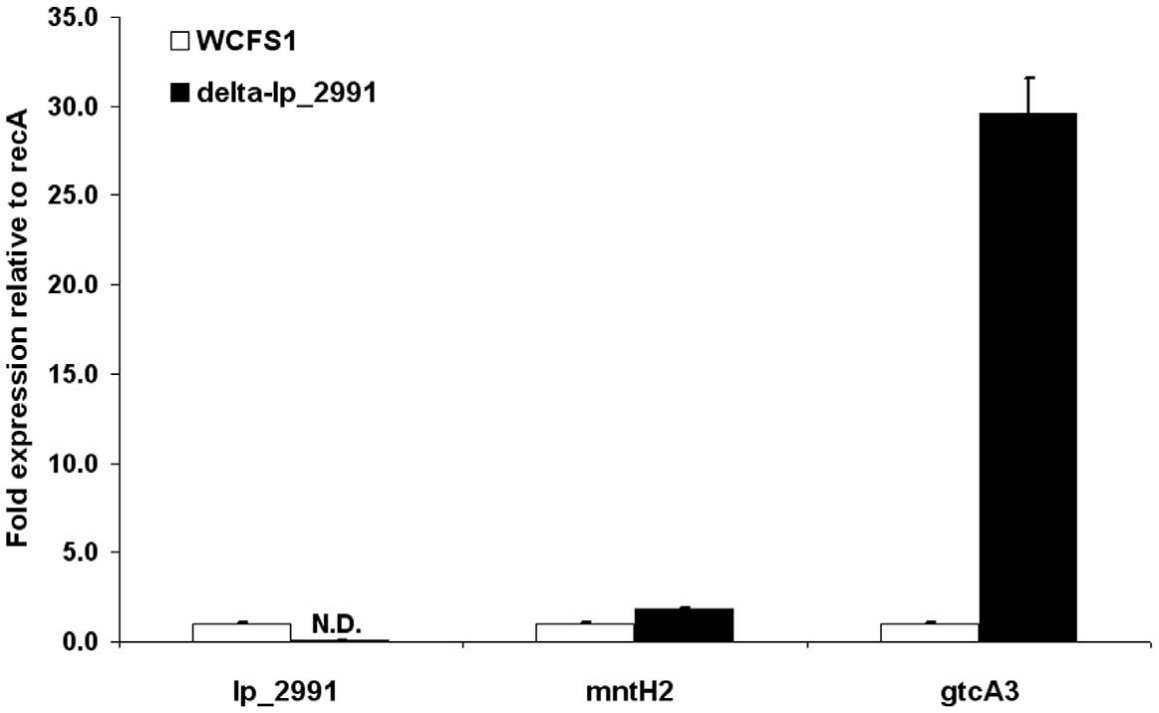
Relative gene expression levels determined by quantitative RT-PCR. RNA prepared from the wild type WCFS1 strain and lp-2991 deletion mutant was used for quantitative RT-PCR. The average *Ct* values observed for the target gene transcripts (lp_2991, mntH2 and gtcA3) were normalized to the average *Ct* values obtained for the reference gene *recA* from the same RNA sample. Two replicates of all samples and primer pairs were included and the experiment was performed in duplicate. Fold changes in transcript level were calculated by using the comparative ΔΔ*Ct* method described by Pfaffl et al. [53].

Quantitative reverse transcription-PCR (RT-PCR) was performed to validate the results of the transcriptome analysis for *mntH2* and *gctA3*A using RNA isolated from the WCFS1 and delta-lp_2991 strains. The specificity of the PCR products was confirmed by a combination of melting curve analysis and 1.5% agarose gel electrophoresis (data not shown). The *gtcA3* gene which lies directly downstream of lp_2991 was indeed strongly up-regulated (29.6 fold) in the mutant strain suggesting that it is normally repressed by lp_2991 under these growth conditions (Fig. 4). In contrast transcript levels of *mntH2*, which is orientated in the opposite direction to lp_2991 were only modestly altered in the deletion mutant (1.8 fold) suggesting that this gene is not directly regulated by the putative transcription regulator. The lp_2991 gene was expressed in strain WCFS1 but no specific PCR product was amplified for in the lp_2991 deletion mutant as expected.

## Discussion

In this study we aimed to determine whether 42 different strains of *L. plantarum*, several strains of which are currently marketed as probiotics possesses different immunomodulatory properties in co-culture with DCs. Additionally, we aimed to identify genetic loci of *L. plantarum* WCFS1, that influence the immune response using comparative genome hybridization data and a gene-trait matching approach. The *L. plantarum* strains differed considerably in their ability to induce pro and anti-inflammatory cytokines. Immune responses to strain NIZO1839 were strikingly lower than for all other strains suggesting that it might directly attenuate immune responses, possess non-typical MAMPs or produce a capsule-polysaccharides that interferes with innate recognition [59]. The amounts of IL-10 induced by the 42 strains ranged from 28 pg/mL to 1095 pg/mL (39 fold) and IL-12 measurements ranged from 20 to 11996 pg/mL (600 fold). The amounts of TNF-alpha induced by the strains ranged from very low (close to the detection limit of 0.7 pg/mL) to more than 8.4 ng/mL. These ranges are higher than that reported for different *Bifidobacterium longum* strains (8-fold) [60] and for multiple *Lactobacillus* and *Bifidobacterium* species in PBMC co-culture assays (10–15 fold) [26], [60], [61], [62], [63]. The highest IL-10 secreting cells in PBMCs are the monocytes which typically represent about 8% of the total leucocytes. This could explain why a larger range of IL-10 measurements were obtained in our experiments using a pure dendritic cell population. The strain differences observed in our experiments were not due to variation in CFU of bacteria as the samples used in the assays were checked twice by plating on solid medium and measurements of optical density (OD_600 nm_).

Probiotics might affect the immune system by the induction of regulatory T cells [64], [65]. Recently a study was published suggesting that probiotics may not directly generate regulatory T cells but induce regulatory DCs via the differentiation of naïve T cells into regulatory T cells in the mesenteric lymph nodes [12]. Regulatory T cells are important in immunological tolerance by the suppression of effector T cells at inflammatory sites. The study by Kwon et al. underlines the important role of dendritic cells in the induction of regulatory T cells and how *in vitro* assays (as described in this study) can be used for the selection of probiotic strains and species that have therapeutic effects in models of inflammatory bowel disease, atopic dermatitis and rheumatoid arthritis.

The large variation in strain immune profiles suggested that there could be some underlying strain-dependent genetic differences influencing the innate response to *L. plantarum*. Therefore results from the cytokine secretion or concentration ratios by the DCs were correlated with the gene presence/absence patterns in the *L. plantarum* strains by regression using the Random Forest algorithm. The output of the algorithm is a model (forest) consisting of many different decision trees. As an output of the algorithm importance measures for each individual gene of the *L. plantarum* WCFS1 genome are given. Genes that score with high importance show a high correlation with the tested immune response and are therefore the most likely candidates to cause the change in immune response. The Random Forest Algorithm model has been used for many applications in bioinformatics, for example to identify single nucleotide polymorphisms predictive of a certain phenotype [66], and to select disease marker genes from microarray gene expression datasets relevant for the prediction of a certain disease [67].

This approach allowed us to identify eight variable *L. plantarum* genes which influence the immune response of DCs to *L. plantarum*. Other genes influencing the immune response of DC may also exist but were not picked up in this screen because a natural mutant was not present in the strain collection. For example, this would include essential genes involved in the production of MAMPs such as peptidoglycan and LTA that form key structural elements of the bacterial the cell wall. Nevertheless, differences in these structures are known to occur due to modifying enzymes which can affect recognition by innate receptors. Point mutations or small deletions that alter bacterial gene expression or protein activity could also modulate the immune response but these would not be detected using this approach. As the genome comparison is based on strain WCFS1, genes present in the other strains but not in WCFS1 may also be involved in immunomodulation. Six of the identified genes were located in operons linked to bacteriocin production or secretion, the other two encode a bile salt hydrolase and a predicted transcriptional regulator of which the exact function is unknown. Deletion mutants of the candidate genes were constructed in the WCFS1 strain in order to validate their anticipated effect on cytokine induction and all but one (the bile salt hydrolase) affected the immune response as predicted by the direction of the correlation. The identification of false positive candidate genes has been reported previously, e.g. only one of the two candidate mannose-specific adhesin genes identified by gene trait matching was shown to be correct [28]. As far as we know this is the first time a comparative genome hybridization approach has been used to identify bacterial gene loci that modulate host immune responses.

Most of the candidate genes influencing the immune response were involved in bacteriocin production and secretion. Bacteriocins are antimicrobial peptides secreted by bacteria that inhibit the growth of closely related micro-organisms. Bacteriocins produced by lactic acid bacteria have received special attention due to their potential use as food preservatives [68]. For example, the bacteriocin called nisin A and nisin-producing strains *Lactococcus lactis* are used commercially worldwide in dairy products, as a bio-preservative [69], [70]. Among the different bacteriocins described in *L. plantarum* strains [56], [71], [72], [73], the complex pln regulon is the best known. This system is organized into five operons (see Fig. 1). The regulatory operon (*plnABCD*) encodes an inducing bacteriocin-like peptide (*plnA*), a histidine protein kinase (HPK) (*plnB*), and two cytoplasmic response regulators (RR) (*plnC* and *plnD*). Another operon (*plnGHSTUVWX*) is associated with plantaricin transport and the operons (*plnJKLR*, *plnMNOP*, *plnEFI*) are related to plantaricin production and immunity [56], [74]. Genes *plnW*, *plnX* and *plnY* are related to membrane integral proteins and other plantaricin biosynthesis proteins. Deletion mutants were constructed for *plnG* and the operons *plnEFI* and *plnGHSTUVWX*. All the deletion mutants of these gene loci induced significantly increased levels of IL-10 secretion (3.3-fold), IL-12p70 (2.4-fold) and TNF-alpha (7.4 fold) compared to the wild-type strain WCFS1. It is possible that the bacteriocin secreted by *L.plantarum* affects the DC immune response because some human antimicrobial peptides have also been shown to activate Toll-like receptors and modulate immune functions [56], [75], [76]. Three independent approaches, namely (*in vivo* expression technology (R-IVET, [77]), qRT-PCR [78] and transcriptomics [79] showed that the expression of the *L. plantarum* plantaricin immunity protein PlnI is induced in the mouse intestine, suggesting that it may be important *in vivo*.

*Lp_2991* was one of the candidate genes identified during the *in silico* gene-trait matching. DC stimulation with the deletion mutant of *lp_2991* led to a significantly higher secretion of IL-10, IL-12p70 and TNF-alpha compared to the wild-type control (Table 3 and Fig. 3). *Lp_2991* encodes for a predicted transcriptional regulator gene upstream of gt*cA3*, a putative teichoic acid glycosylation protein. Upstream of *lp_2991* is a manganese transport gene *mntH2* which is orientated in the opposite direction.

Transcriptome analysis and qPCR data showed that transcript level of *gtcA3* was substantially increased in the *lp_2991* deletion mutant (44 and 29 fold respectively). This supports the idea that *lp_2991* is a repressor of *gtcA3* transcription and points to this enzyme as being a prime candidate for the altered immune response. GtcA3 is predicted to glycosylate teichoic acid including lipoteichoic acid (LTA) which is a known TLR2 agonist capable of modulating immune cell responses. Modification of LTA e.g. by substitution can have striking effects on the immune response as shown by Grangette et al., using a mutant in which *dltB* was deleted [30]. *DltB* is a putative transmembrane protein predicted to be involved in the passage of the activated d-alanyl-Dcp complex across the glycerol phosphate backbone of LTA. The *dltB* mutant of *L. plantarum* increased IL-10 secretion and dramatically raised the IL-10/IL-12 ratio in PBMC co-culture assays. In our experiments the *lp_2991* deletion mutant also led to an increased IL-10 secretion in immune assays (6.3 fold) compared to wild type strain but the IL-10/IL-12 ratio was only slightly elevated due a corresponding increase in the induced levels of IL-12 (3.2-fold).

As the transcript levels of *gtcA3* were increased 44 fold in the *lp_2991* deletion mutant and this enzyme is predicted to glycosylate TA or LTA we consider it most likely to be responsible for the altered immune response. However, we cannot rule out possible effects due to changes in *mntH2* gene expression which was modestly affected in the mutant (1.8 and 2.4 fold increase by qPCR and transcriptome analysis respectively). Manganese is an important element involved in the protection of *L. plantarum* against oxidative stress [80]. One uptake mechanism described is the Mn (2+) and Cd (2+)-specific P-type ATPase MntA. Besides *mntA*, WCFS1 encodes an ABC transporter system (*mtsCBA*) and three genes encoding Nramp transporters (*mntH1*, *mntH2* and *mntH3*)[80]. Studies conducted in pathogenic bacteria have shown that Nramp transporter mutants are less virulent [81] due to the role of Nramp in sequestering of Mn(2+) which is a cofactor for enzymes that protect against host oxidative killing mechanisms. The mutation of *lp_2991* may increase manganese transport and therefore enhance survival of lactobacilli in the phagolysosome. This may alter the kinetics and magnitude of the immune response due to altered release of ligands for host pattern recognition receptors.

This study emphasizes the usefulness of *in silico* gene-trait matching in assessing the role of specific bacterial genes in the interaction with the host immune system, an approach that is fully supported by the recent availability of full genome sequences for some lactobacilli. Screening with another *L. plantarum* than WCFS1 may also lead to the identification of other genes influencing the immune response. In the future this knowledge may be useful to select probiotic strains with anti-inflammatory or immune stimulatory properties. Future work is aimed at understanding the role of the genes we have identified in modulating the immune response to *L. plantarum*.

## Supporting Information

Figure S1 CD83 and CD86 expression by monocyte-derived dendritic cells derived from blood of five different donors after stimulation with 20 different *L. plantarum* strains. Each symbol represents a different *L. plantarum* strain.
(2.14 MB TIF)

Table S1 Origin of bacterial strains used in this study.
(0.07 MB DOC)

Table S2 Transcriptome analysis of WCFS1 and lp_2991 deletion mutant.
(0.05 MB PDF)
