# Supplementary figures and images for: Identification of Genetic Loci in Lactobacillus plantarum That Modulate the Immune Response of Dendritic Cells Using Comparative Genome Hybridization
Source: PLoS One. 2010 May 13;5(5):e10632. doi: 10.1371/journal.pone.0010632 (PMC2869364; doi:10.1371/journal.pone.0010632)

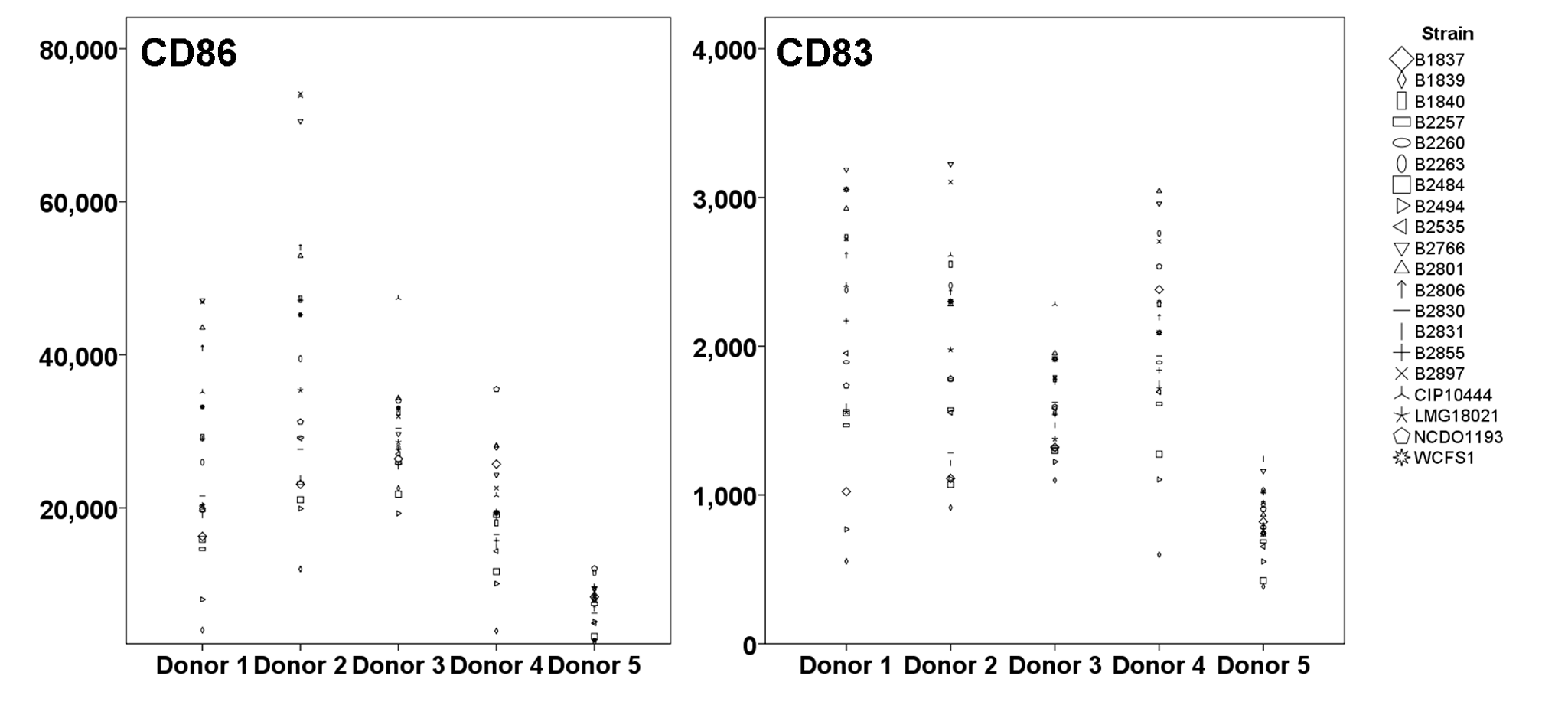

Supplement: Figure S1 — CD83 and CD86 expression by monocyte-derived dendritic cells derived from blood of five different donors after stimulation with 20 different L. plantarum strains. Each symbol represents a different L. plantarum strain. (2.14 MB TIF) [file pone.0010632.s001.tif]
