# Supplementary material for: Identification of Genetic Loci in Lactobacillus plantarum That Modulate the Immune Response of Dendritic Cells Using Comparative Genome Hybridization
Source: PLoS One. 2010 May 13;5(5):e10632. doi: 10.1371/journal.pone.0010632 (PMC2869364; doi:10.1371/journal.pone.0010632)
Supplement: Table S1 — Origin of bacterial strains used in this study. (0.07 MB DOC) [file pone.0010632.s002.doc]

| **Strain #** | **Received as** | **Isolation source** | **Geographical origin** |
| --- | --- | --- | --- |
| **NIZO1836** | **WCFS1** | Human saliva | England |
| **NIZO2263** | **LP80** | Silage | n.a. |
| NIZO2814 | Lp95 | Wine red grapes | Italy |
| **CIP102359** | **CIP102359** | Human spinal fluid | France |
| **NIZO2726** | **ATCC8014** | Maize ensilage | n.a. |
| NIZO2891 | LD3 | Radish pickled | Vietnam |
| NIZO2457 | CHEO3 | Pork pickled sour sausage | Vietnam |
| NIZO2535 | LD2 | Orange fermented | Vietnam |
| NIZO2830 | BLL(EI31) | n.a. | n.a. |
| **NIZO2259** | **CIP104452** | Human tooth abscess | France |
| NIZO2831 | CECT221(24Ab04) | Grass silage | United States |
| **NIZO2262** | **LM3** | Silage | n.a. |
| NIZO2494 | NCTH27 | Pork pickled sour sausage | Vietnam |
| **NCDO1193** | **NCDO1193** | Vegetables | n.a. |
| NIZO2806 | LMG9208 | Sauerkraut | United Kingdom |
| **NIZO2896** | **ATCC14917^a^** | Cabbage pickled | Denmark |
| NIZO2741 | NOS140 | Cabbage kimchi | Japan |
| **NIZO1837** | **299** | Human colon | United Kingdom |
| NIZO2855 | N58 | Pork pickled sour sausage | Vietnam |
| NIZO2877 | X17 | Hot dog | Vietnam |
| **NIZO2260** | **299v/DSM9843** | Human intestine | United Kingdom |
| NIZO2029 | MLC43 | Raw cheese with rennet | Italy |
| NIZO2889 | LAC7 | Banana fermented | Vietnam |
| **NIZO2264** | **LP85-2^b^** | Silage | France |
| NIZO2484 | NCTH19-1 | Pork pickled sour sausage | Vietnam |
| NIZO2485 | NCTH19-2 | Pork pickled sour sausage | Vietnam |
| **NIZO2261** | **NC8** | Grass silage | Sweden |
| NIZO2802 | KOG24 | Cheese | Japan |
| NIZO2801 | KOG18 | Turnip pickled | Japan |
| **NIZO3400** | **LMG18021** | Milk | Senegal |
| NIZO2753 | Q2 | Sourdough fermented | Italy |
| **NIZO1839** | **SF2A35B ^b^** | Sour cassava | South America |
| **NIZO2258** | **CIP104451** | Human urine | France |
| **NIZO2257** | **CIP104450** | Human stool | France |
| **CIP104448** | **CIP104448** | Human stool | France |
| NIZO2897 | DKO22 **^b^** | Sour cassava | Nigeria |
| NIZO2766 | H14 | Sourdough fermented | Italy |
| NIZO2757 | H4 | Sourdough fermented | Italy |
| NIZO2776 | CECT4645 | Cheese | n.a.. |
| **NIZO2256** | **CIP104441** | Human stool | France |
| **NIZO1838** | **CIP104440** | Human stool | France |
| **NIZO1840** | **NCIMB12120 ^b^** | Cereal fermented (Ogi) | Nigeria |

n.a. not available

Strains in bold were also compared in Molenaar et al (2005). The other strains were new in this study.

^a^ Draft genome sequence available April 2009 (NZ_ACGZ00000000.1)

^b^ Putative subspecies *argentoratensis*
