# Supplementary material for: Identification of Genetic Loci in Lactobacillus plantarum That Modulate the Immune Response of Dendritic Cells Using Comparative Genome Hybridization
Source: PLoS One. 2010 May 13;5(5):e10632. doi: 10.1371/journal.pone.0010632 (PMC2869364; doi:10.1371/journal.pone.0010632)
Supplement: Table S2 — Transcriptome analysis of WCFS1 and lp_2991 deletion mutant. (0.05 MB PDF) [file pone.0010632.s003.pdf]

Gene no. (gene name)	Fold change		Description of proposed function
	**Genes with increased relative transcript levels in lp_2991 deletion mutant compared to WCFS1**	**FDR**	
**Cell envelope**			
lp_2988 (zmp3)	2.2	2.70E-05	extracellular zinc metalloproteinase, M10 family (putative)
lp_2809	2.5	9.89E-03	extracellular protein (putative)
lp_0730 (tagO)	4.4	3.37E-05	undecaprenyl-phosphate N-acetyl-glucosaminyl transferase
lp_2989 (gtcA3)	45	1.83E-07	teichoic acid glycosylation protein (putative)
**Cellular processes**			
lp_0214	3.1	6.66E-03	chromosome condensation protein (putative)
**Central intermediary metabolism**			
lp_1173	5.6	1.19E-04	UDP-N-acetylglucosamine 2-epimerase
lp_0181 (mapB)	5.9	3.76E-02	maltose phosphorylase
**Energy metabolism**			
lp_2659 (xpkA)	2.1	3.31E-04	xylulose-5-P phosphoketolase & fructose-6-P phosphoketolase
lp_3490	8.6	1.58E-02	FMN-binding protein
**Hypothetical proteins**			
lp_1726	2.1	2.97E-02	unknown
lp_0311	2.2	1.67E-02	acetyltransferase (putative)
lp_2066	2.2	3.76E-02	unknown
lp_3346	2.9	2.87E-02	unknown
lp_3002	3.2	3.18E-03	integral membrane protein
lp_2230	16.7	1.70E-07	unknown
lp_2093	18.1	1.22E-02	unknown

lp_3348	19.1	1.41E-06	unknown
**Protein synthesis**			
lp_2807 (tyrS)	2.8	3.83E-02	tyrosine--tRNA ligase
**Purines, pyrimidines, nucleosides and nucleotides**			
lp_2702 (pyrC)	4.1	3.21E-03	dihydroorotase
lp_2699 (pyrD)	4.9	4.28E-04	dihydroorotate oxidase
lp_2701 (pyrAA)	5.6	1.00E-04	carbamoyl-phosphate synthase, pyrimidine-specific, small chain
lp_2700 (pyrAB)	6.8	2.71E-05	carbamoyl-phosphate synthase, pyrimidine-specific, large chain
lp_2698 (pyrF)	7	1.03E-05	orotidine-5'-phosphate decarboxylase
lp_2697 (pyrE)	17	1.76E-07	orotate phosphoribosyltransferase
**Regulatory functions**			
lp_1938	2.2	2.80E-02	transcription regulator, LysR family
lp_2704 (purR1)	2.6	2.71E-05	pyrimidine operon regulator
**Transport and binding proteins**			
lp_2992 (mntH2)	2.4	1.23E-02	manganese transport protein
lp_0092	2.5	3.46E-03	ABC transporter, substrate binding protein, oligopeptide
lp_2371 (pyrP)	2.6	9.81E-07	uracil transport protein
lp_0286 (pts6C)	5.1	3.39E-03	cellobiose PTS, EIIC
lp_1792	19.7	1.43E-02	ABC transporter, permease protein
lp_p3_38	3.2	1.10E-03	nickase

Genes with decreased relative transcript levels in lp_2991 deletion mutant compared to WCFS1		FDR	
**Biosynthesis of cofactors, prosthetic groups, and carriers**			
lp_0369 (gshR1)	-5.5	1.53E-05	glutathione reductase
**Cellular processes**			
lp_2210 (ftsK2)	-5.8	4.20E-02	cell division protein FtsK
**DNA metabolism**			
lp_1839 (parC)	-2.3	1.51E-05	topoisomerase IV, subunit A
**Energy metabolism**			
lp_3595 (rhaB)	-5.2	3.32E-07	rhamnulokinase
lp_3449 (nox5)	-2.5	4.42E-02	NADH oxidase
**Hypothetical proteins**			
lp_1533	-2.9	3.79E-05	methyltransferase (putative)
lp_0753	-2.8	2.71E-04	integral membrane protein
lp_0967	-2.8	2.19E-02	unknown
lp_2058	-2.4	8.25E-03	endonuclease (putative)
lp_1390	-2.2	4.40E-03	acetyltransferase, GNAT family (putative)
lp_1136	2	1.45E-04	oxidoreductase, NAD(P)-dependent
lp_2114	2	1.07E-03	NTP pyrophosphohydrolase (putative)
**Protein synthesis**			
lp_0443 (dus1)	-2.3	9.88E-04	tRNA-dihydrouridine synthase

**Regulatory functions**			
lp_0188 (scrR)	-3.4	1.12E-03	oligosucrose operon repressor
lp_0319	-2.3	4.29E-02	transcription regulator, spermidine/putrescine transport operon
**Transport and binding proteins**			
lp_2531 (pts18CBA)	-13.1	9.98E-04	N-acetylglucosamine and glucose PTS, EIICBA
lp_3686	-9	1.21E-03	ABC transporter, substrate binding protein
lp_0317 (potB)	-5.1	3.12E-02	spermidine/putrescine ABC transporter, permease protein
lp_0218	-4.9	1.89E-03	ABC transporter, ATP-binding protein
lp_2352	-3	5.98E-06	ABC transporter, ATP binding binding protein, D-Methionine -like precursor
lp_3279 (kup2)	-2.5	6.55E-03	potassium uptake protein
lp_2351	-2.4	3.26E-03	ABC transporter, permease protein, D-Methionine -like precursor
lp_0367 (choS)	-2.3	3.29E-05	glycine betaine/carnitine/choline ABC transporter, substrate binding and permease protein
lp_0368 (choQ)	-2.3	1.12E-04	glycine betaine/carnitine/choline ABC transporter, ATP-binding protein
